# Modular cytosine base editing promotes epigenomic and genomic modifications

**DOI:** 10.1093/nar/gkad1118

**Published:** 2023-11-22

**Authors:** Julian Weischedel, Laurence Higgins, Sally Rogers, Anna Gramalla-Schmitz, Paulina Wyrzykowska, Simone Borgoni, Thomas MacCarthy, Richard Chahwan

**Affiliations:** Institute of Experimental Immunology, University of Zurich, Zurich 8057, Switzerland; Living Systems Institute, University of Exeter, Exeter EX4 4QD, UK; Living Systems Institute, University of Exeter, Exeter EX4 4QD, UK; Institute of Experimental Immunology, University of Zurich, Zurich 8057, Switzerland; Institute of Experimental Immunology, University of Zurich, Zurich 8057, Switzerland; Institute of Experimental Immunology, University of Zurich, Zurich 8057, Switzerland; Department of Applied Mathematics & Statistics, Stony Brook University, NY 11794-3600, USA; Institute of Experimental Immunology, University of Zurich, Zurich 8057, Switzerland

## Abstract

Prokaryotic and eukaryotic adaptive immunity differ considerably. Yet, their fundamental mechanisms of gene editing via Cas9 and activation-induced deaminase (AID), respectively, can be conveniently complimentary. Cas9 is an RNA targeted dual nuclease expressed in several bacterial species. AID is a cytosine deaminase expressed in germinal centre B cells to mediate genomic antibody diversification. AID can also mediate epigenomic reprogramming via active DNA demethylation. It is known that sequence motifs, nucleic acid structures, and associated co-factors affect AID activity. But despite repeated attempts, deciphering AID’s intrinsic catalytic activities and harnessing its targeted recruitment to DNA is still intractable. Even recent cytosine base editors are unable to fully recapitulate AID’s genomic and epigenomic editing properties. Here, we describe the first instance of a modular AID-based editor that recapitulates the full spectrum of genomic and epigenomic editing activity. Our ‘Swiss army knife’ toolbox will help better understand AID biology per se as well as improve targeted genomic and epigenomic editing.

## Graphical Abstract

**Graphical Abstract:**
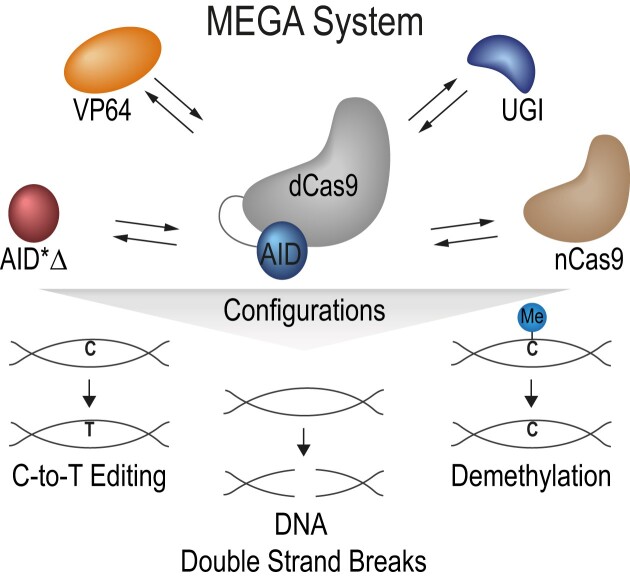

## Introduction

The discovery of the RNA-guided Clustered Regularly Interspaced Short Palindromic Repeats (CRISPR) and the CRISPR associated nuclease 9 (Cas9) system has ushered in a new era of genetic engineering (1,2). CRISPR-Cas9 originates from the prokaryotic adaptive immune system against exogenous double stranded DNA (3). Despite being a promising tool, predicting and controlling the editing outcome presented various challenges. In most cases, Cas9-induced DNA double strand breaks (DSBs) are resolved by error-prone non-homologous end joining, leading to insertions and deletions (Indels) at the break site. The random nature of this process creates the risk of unwanted genomic outcomes (4).

Genomic editing plays a crucial role in vertebrate adaptive immunity, too. For example, B cells rely on such processes to diversify their immunoglobulin affinity and mediate B cell maturation. This ensures adequate protection against the vast plethora of pathogenic threats (5,6). Antibody affinity maturation is mediated by the activation-induced deaminase (AID) protein through the process of somatic hypermutation (SHM) and class swich recombination (CSR) (7). By deaminating cytosine (C) to uracil (U) in single stranded DNA (ssDNA) AID creates an uracil guanosine (G) mismatch (8). Either through replication or error-prone DNA repair, the mismatch resolves in a fixed C to thymine (T) transition mutation (9). AID targets preferentially C’s within WRC and particularly in WGCW overlapping hotspots (OHS) (W = adenosine (A) or T, R = A or G, Y = C or T). SYC motifs (S = G or C) are inert coldspots where C’s are less likely to undergo mutations. (10–12). During SHM, single point mutations occur largely within the complementarity determining regions (CDRs) of the antibody variable heavy and light chain (13). Interestingly, SHM has not yet been recapitulated completely ex vivo. Besides its established genomic activity, AID has also been associated with active epigenetic editing. AID-dependent deamination of 5-methylcytosine (5mC) creates a T:G mismatch, which eventually is replaced with a non-methylated C through error-free base excision repair (14,15). Under *in vitro* conditions, wild type AID has clearly shown to be able to recognise and deaminate 5mC (16,17). In vivo studies, however, suggest AID’s role in demethylation might be locus restricted during B cell development (18,19). Taken together, AID is a multifunctional mutator protein with a two-tier activity spectrum.

Cytosine base editors (CBEs) are an improvement of the prokaryotic CRISPR/Cas system. They combine the catalytic activity of a vertebrate cytosine deaminase with the gene targeting ability of a nuclease-deficient prokaryotic Cas complexed via a guide RNA (gRNA) (20–22). In some sense, CBEs represent a hybrid prokaryotic-eukaryotic adaptive immunity. While CRISPR introduces precise DSBs, CBEs lead to targeted single nucleotide mutations (3,20). As base editing does not disrupt the genome integrity it is less prone to unpredictable and potentially deleterious Indels at the targeted site. Thus far, CBEs development focused on the mutagenesis of single nucleotides. Even though there are base editors deploying AID or orthologs of it, they are not able to exploit AID’s full functional potential (21–26). In particular, AID’s epigenomic potential is highly underdeveloped.

In this study, we describe a novel human AID-focused modular CBE that allows both targeted genomic and epigenomic editing through cytosine deamination. Our Modular Epigenomic and Genomic AID base editor system (MEGA) takes advantage of AID’s multifunctional characteristics to induce targeted C-to-T mutations, DSBs, and 5mC demethylation. The respective effects are defined by distinct MEGA configurations. To our knowledge, this functional variability has not been reported within an integrated system, yet (27). Hence, we provide a new and better equipped programmable ‘Swiss army knife’ editing toolkit. Its use will potentially help to improve our current understanding of AID’s molecular function and allow us to fully translate at high resolution AID activity *ex vivo*.

## Materials and methods

### Construct cloning and additional cytosine base editors

MEGA-2 was assembled by introducing AID*Δ-XTEN-Linker at the N-terminus of dCas9-VP64 in the backbone vector Cas9m4VP64 (Addgene #47319) through two-step ligation. AID*Δ was amplified from the pGH335_MS2-AID*Δ-Hygro plasmid (Addgene #85406) with primers including the XTEN-Linker at the C-terminus. For MEGA-1 the same cloning strategy was used but with human full-length wild type AID. MEGA-3 was constructed in a one-step ligation process whereby AID*Δ-XTEN was introduced into the Cas9m2 vector (Addgene #47317). MEGA-4 was cloned in the same way as MEGA-3 but using the hCas9_D10A (Addgene #41816) backbone instead. Cytosine base editors AID-BE3 (Addgene #100803) , BE4max (Addgene #112093) and CP1012 CBEmax (Addgene #119801) were purchased through Addgene.

### gRNA plasmid constructs

Specific gRNAs targeting the *GFP*, *TP53BP1* and *CH12-F3 IgH variable domain* locus were designed by manual curation or using CRISPRdirect (28). For *ATP1a1* and *mouse MyoD* published gRNAs were used (29,30). Cloning of gRNA expressing vectors was done as described previously (31). In brief, 19 bp of the respective gRNA sequence were incorporated into two 60mer oligo nucleotides. The two oligos were annealed and extended using Phusion polymerase (NEB®). Eventually, the destination plasmid gRNA_Cloning vector (Addgene #41824) was linearized with AflII and the 100 bp fragment was incorporated by Gibson assembly. A complete list of gRNAs is in Supplementary Table S1.

### Cell culture

All cells were maintained in 10 cm dishes (Sarstedt) at 37°C and 5% CO_2_. HEK293A, HEK293T-GFP and 3T3 cells were grown in DMEM (Sarstedt) supplemented with 10% FBS (Sarstedt), 1% penicillin/streptomycin + l-glutamine (Gibco), 1% sodium pyruvate (Gibco) and 50 μM β-mercaptoethanol (Gibco). CH12-F3 mouse erythroleukemia B cells were grown in RPMI 1640 (Sarstedt) supplemented with 10% FBS (Sarstedt), 1% penicillin/streptomycin + l-glutamine (Gibco), 1% sodium pyruvate (Gibco), 5% NCTC-109 (Gibco) and 50 μM β-mercaptoethanol (Gibco).

### Lipid-based cell transfection

Lipid-based transfections were done with Lipofectamine™ 3000 (Invitrogen) or jetPrime® (Polyplus). Manufacturers protocols were followed. In brief, for Lipofectamine™ 3000 transfections, 0.5 × 10^6^ HEK293T-GFP or 1 × 10^6^ 3T3 cells/well were seeded in 6-well plates. 750 ng of MEGA or wild type Cas9 plasmid, 500 ng of gRNA plasmid and if required 500 ng of UGI plasmid were mixed with 5 μl P3000™ reagent and 3.75 μl Lipofectamine™ 3000 reagent. When using jetPrime® 0.1 × 10^6^ HEK293T-GFP or 3T3 cells/well were seeded in 12-well plates. In total 300 ng MEGA plasmid was combined with 200 ng gRNA plasmid and if required with 200 ng UGI plasmid. The DNA was mixed with jetPrime® buffer and jetPrime® reagent (1:2 ratio DNA-to-reagent). Four hours after transfection cell media was changed. After 72 h, cells were harvested and either directly analysed or stored at –80°C for sequencing.

### Cell electroporation

To mutate the murine variable heavy chain domain CH12-F3 cells were electroporated using the Gene Pulser Xcell Eukaryotic System (BioRad). In brief, 5 × 10^6^ cells were resuspended in Opti-MEM medium (Gibco) together with 3 μg DNA (1000 ng MEGA plasmid and 500 ng of each respective gRNA plasmid) in 0.4 cm gap cuvettes (BioRad). Electroporation was done with 30 ms pulse and square wave setting. For electroporation of 3T3 cells the SF Cell Line 4D-Nucleofector™ X Kit S (Lonza) was used with program EN-158. 1 × 10^6^ cells were resuspended with 750 ng MEGA plasmid and 1000 ng mouse MyoD gRNA plasmid.

### Flow cytometry

Cells were harvested and washed with FACS buffer (1× PBS with 2% BSA and 1 mM EDTA) at 400 g at 4°C for 4 min. For live/dead discrimination cells were incubated with PI. GFP fluorescence was detected using a SONY SP6800 spectral analyser or BD FACS Fortessa. GFP-negative HEK293A and GFP-positive HEK293T-GFP cells were used as negative and positive control, respectively. Loss in GFP signal was compared to non-transfected HEK293T-GFP cells using FlowJo V10.

### PacBio long-read single molecule sequencing of the GFP locus

For long-read single molecule sequencing we used the Pacific Biosciences (PacBio) Sequel II sequencer with the 8M SMRTcell and a 15 h movie. Genomic DNA was isolated with the GeneJet Genomic DNA extraction kit (Thermo Fisher Scientific) from frozen cells which were previously transfected with MEGA and gRNAs. Library preparation of the *GFP* amplicon was done in a one-step PCR reaction. The respective primers contained a PAD sequence for ligation to the SMRTcell, a barcode sequence for multiplexing and the GFP-specific sequence. Primer sequences are listed in Supplementary Table S2. To avoid PCR bias three independent PCR reactions for each sample were combined. Eventually, all barcoded amplicons were pooled in an equimolar ratio and cleaned with GeneJet PCR Purification Kit (Thermo Fisher Scientific). Follow up AMPure PB bead clean-up, adaptor ligation and sequencing were done by the Functional Genomic Centre Zurich.

### Illumina sequencing of GFP, ATP1a1, TP53BP1, variable heavy chain domain and MyoD

Illumina amplicon library preparation was done with the Nextera XT DNA Library Preparation Kit (Illumina). PAD sequences for Nextera adaptors were added to the locus specific primers and subsequently each locus was amplified (Supplementary Table S2). Primers are listed in Supplementary Table S3. Three independent reactions were performed and pooled for each sample to avoid PCR bias. After PCR clean-up Nextera Index were added to the amplicons through a second PCR reaction. PCR products’ quantity and quality were assessed using the Qubit Fluorometer (Thermo Fisher Scientific) and TapeStation (Agilent). Samples were sequenced by the Exeter Sequencing Service with an Illumina HiSeq Sequencer.

### Bisulfite treatment and sequencing

Bisulfite conversion of DNA from 5 × 10^4^ cells was performed using the EZ DNA methylation kit (Zymo Research). In brief, the genomic DNA was incubated with CT conversion reagent at 98°C for 8 mins, then 14 cycles of 95°C for 15 s and 64°C for 15 min. DNA was cleaned and eluted following the manufacturer's instructions. Subsequently, the target region of the DMR5 MyoD enhancer region was amplified by PCR at 95°C for 12 min, then 40 cycles of 95°C for 90 s, 58°C for 90 s, 72°C for 45 s. A final elongation step of 10 min was included in all reactions. PCR products were analysed by gel electrophoresis and products purified with MiniElute (Qiagen). Three separate PCRs were performed on each sample to control for PCR bias in the subsequent analysis. PCR products were pooled from individual samples and cloned into a TA vector and sequenced by Sanger sequencing. Only unique sequences (as determined by either unique CpG methylation pattern or unique non-conversion of non-CpG cytosines) are shown, and all sequences had a conversion rate > 99%.

### RNA isolation and RT qPCR

RNA was isolated and purified using the RNeasy Mini Kit (Qiagen) according to manufacturer's instructions. RNA concentration was measured with NanoDrop-ND 1000 (Thermo Fisher Scientific) and cDNA was synthesized using the High-Capacity cDNA Reverse Transcription Kit (Thermo Fischer Scientific). Real time quantitative PCR (RT qPCR) for target genes was performed using either HOT FIREPol EvaGreen qPCR Mix Plus w/o ROX (Solis BioDyne) or Maxima SYBR Green/Fluorescein qPCR Master Mix 2× (Thermo Fisher) with CFX384 Touch Real-Time PCR System (Bio-Rad). Input cDNA corresponded to 30 ng total RNA. Respective primers are listed in Supplementary Table S4. Pre-analysis was done with the CFX Maestro Software and the relative expression values were calculated with the ΔΔCt method, normalizing the Ct values to the housekeeping gene *Actin B* (mouse).

### Targeted amplicon sequencing analysis

Demultiplexed amplicon deep sequencing data were analysed with CRISPResso2 web version (32). PacBio Sequencing data were uploaded as single end and Illumina Sequencing data as paired end reads. Minimum homology for alignment was set to 80%. Remaining parameters were kept at default. Nextera PE was chosen for adapter trimming of Illumina-derived deep sequencing data. Heatmaps were generated with CRISPResso2 output file ‘Nucleotide_percentage_table.txt’. For targeted base editing, base editing window and base editing purity ‘Selected nucleotide percentage table around gRNA.txt’ was used. Indel and substitution frequency was calculated with ‘CRISPResso quantification of editing frequency.txt’ files. Sequence histograms were done with the ‘Modification_Count_Vectors.txt’ files. The mean percentage value was calculated for samples with more than one repeat. Subsequent values of non-transfected controls were subtracted from sample values for normalisation. Graphs were made with GraphPad Prism V7.

### SHMprep mutation analysis of immunoglobulin locus

Raw FASTQ data (R1 and R2) of untreated and treated CH12-F3 cells were processed as previously described (33). In brief, SHMprep was run with default parameters. The joined (R1 and R2) sequences were extracted from the detailed output files (with extension .details) and converted to FASTA format using a custom Python script that included a filter removing sequences without at least 80% similarity to the consensus (unmutated) sequence. The resulting sequences from each sample were compared to the consensus sequence to compute site-by-site mutation frequencies, using a custom R script. Eventually, the mutation frequency of untreated samples was subtracted from the MEGA-4 or wild type Cas9 treated samples. The example primary mouse data used was downloaded from the Observed Antibody Space (OAS) database (dataset ID ERR49859), and the subset of sequences with assigned germline IGHV1-53*01 were selected using a custom shell script. Mutation frequencies were then calculated and plotted as above.

### Microhomology analysis

For the deletion analysis, sequences that were shorter than germline were selected, then aligned to the consensus sequence using the R Biostrings function pairwise alignment. Only sequences containing a single deletion and no insertions were selected for further analysis. Following (34), the deletion was compared to the adjacent sequence to calculate the microhomology length.

### Statistical analysis

For statistical analysis, either an unpaired *t*-test or one way-ANOVA with multiple comparison was used. Standard deviation is always shown for mean values of two to three technical repeats. Calculations and visualisations were performed using GraphPad V7.

## Results

### Transcription activator does not improve AID activity

**Figure 1.**
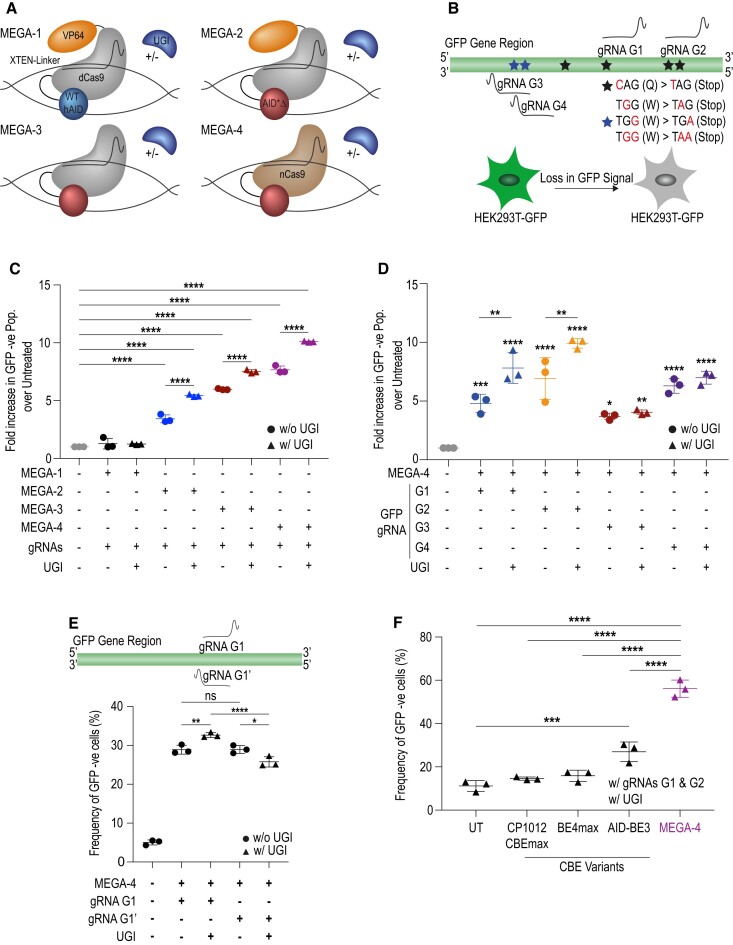
Loss in GFP improves depending on MEGA configuration. (**A**) Schematic representation of MEGA constructs. (**B**) (Upper panel) GFP-specific gRNAs were designed to span sequences where targeted C/G-to-T/A mutations create in-frame stop codons. (Lower panel) Successful editing leads to a loss in GFP fluorescence in HEK293T-GFP cells. (**C**) MEGA-1, -2, -3 and -4 dependent loss in GFP signal when using gRNA G1 and G2 simultaneously. Experiments were done with and without co-expression of UGI. (**D**) Editing of four different *GFP* loci resulted in varying levels of GFP loss. UGI enhanced the phenotype. (**E**) (Upper panel) Position of gRNA G1’ in relation to the position of gRNA G1. (Lower Panel) In the GFP disruption assay no strand bias was seen in the absence of UGI. Adding UGI showed a discrepancy in GFP disruption efficacy. (**F**) Comparing the efficacy to disrupt the GFP signal by MEGA-4 and three previously published CBEs. All results are normalized and shown as fold increase of GFP-negative population over non-transfected control HEK293T-GFP cells. Mean with standard deviation of three independent experiments is shown. Each data point represents one experiment. Three technical repeats were done per experiment. Statistical significance was calculated by a one-way ANOVA. WT hAID (wild type human Activation Induced Deaminase), AID*Δ (hyperactive truncated form of human AID), dCas9 (nuclease dead Cas9), UGI (Uracil Glycosylase Inhibitor). (* *P* ≤ 0.5, ** *P* ≤ 0.001, *** *P* ≤ 0.0001 and **** *P* ≤ 0.00001).

To establish a modular base editing system, we selectively exploited the benefits of prokaryotic and eukaryotic adaptive immunity by merging the Cas9 and AID gene editors’ advantages, respectively. For MEGA-1, we fused full length wild type human AID with a flexible XTEN-Linker to the N-terminus of the *S. pyogenes*-derived nuclease dead Cas9 (dCas9) (Figure 1A, upper left). AID requires ssDNA as substrate for deamination. By adding the virus-derived transcription activator VP64 to the C-terminus of our construct we aimed to increase ssDNA substrate accessibility (Figure 1A). We also complemented our MEGA system with the uracil glycosylase inhibitor (UGI) as an independent co-factor. Inhibition of the endogenous uracil DNA glycosylase with UGI, either directly linked to or co-expressed with the base editor has been shown to improve C-to-T base editing (35). For proof-of-function, we performed a GFP disruption assay in a HEK293T-GFP cell line constitutively expressing GFP. We designed two gRNAs (G1 and G2) to target two *GFP* loci where C-to-T mutations would create premature stop codons (Figure 1B). The change in GFP fluorescence was detected by flow cytometry (Figure 1C and Supplementary Figure S1). Compared to untreated cells MEGA-1 together with a combination of gRNA G1 and G2, to maximize targeting, did not significantly increase the GFP-negative cell population. Adding UGI expression did not show a beneficial effect on GFP loss either (Figure 1C).

### MEGA configurations differentially affect loss of GFP fluorescence

Since MEGA-1 did not improve AID activity, three additional MEGA configurations were constructed to better understand the mode of action of our system. The modular setup allowed us to change the deaminase moiety, remove or retain the transcription activator VP64, and compare nickase Cas9 (nCas9) with dCas9 (Figure 1A). All new configurations made use of AID*Δ a hyperactive mutant of human AID with enhanced cytosine deamination activity (23). The rationale being that AID*Δ might synergise better with our MEGA architecture. All new configurations were phenotypically evaluated as before. Interestingly, a significant increase in GFP-negative cells was detected in all three new MEGA variants (Figure 1C); and the construct architecture differentially affected editing activity. MEGA-2, which contains VP64, increased the GFP-negative population by 3.43 ± 0.34-fold compared to untreated cells. When adding UGI it led to a 5.44 ± 0.1-fold change. MEGA-3, lacking VP64 and without UGI, performed better than MEGA-2 and achieved an increased loss by 5.98 ± 0.05-fold. Again, co-expression of UGI had a beneficial effect and resulted in a 7.52 ± 0.15-fold change. Among our different constructs, the most potent base editor was MEGA-4 using nCas9 and lacking VP64. Many current CBEs use nCas9, which cuts the DNA strand complementary to the gRNA. The non-edited cut strand will be targeted for DNA repair whereby the mutated strand will serve as a template. This improves C/G-to-T/A editing efficacy (20). We detected a 7.67 ± 0.33-fold loss without UGI and 10.11 ± 0.05-fold loss with UGI, respectively (Figure 1C). With gRNA G1 and G2 we edited the positive strand of GFP. To exclude potential strand bias, we repeated the GFP disruption assay with gRNAs targeting the opposite strand (Figure 1B). We did not observe a significant difference in overall GFP loss when using gRNA G3 and G4 compared to gRNA G1 and G2 (Supplementary Figure S2). To further evaluate the activity of MEGA-4 we transfected HEK293T-GFP cells with MEGA-4 and each GFP-specific gRNA separately (Figure 1D and Supplementary Figure S3). Depending on the targeted locus the GFP-loss cell population significantly increased from 3.7 ± 0.27- to 6.92 ± 1.79-fold without UGI and from 4.04 ± 0.2 to 9.92 ± 0.41-fold with UGI, respectively (Figure 1D). Generally, UGI displayed a marked benefit for the positive strand targeting and less so for the negative strand (Figure 1D). The same was observed when using gRNA G1’, which was placed directly opposite to G1 and did not cover any premature stop codons (Figure 1E). Without co-transfecting UGI, gRNAs G1 and G1’ led to a similar increase in the frequency of GFP-negative cells (Figure 1E). When supplementing MEGA-4 and gRNA G1 with UGI, even more cells lost GFP. Adding UGI to MEGA-4 and gRNA G1’, however, resulted in a marked reduction of GFP-negative cells. We compared MEGA-4 to three recently published cytosine base editors, that link either rat APOBEC1 or human wild-type AID to standard or a circularly permutated form of nCas9 (Figure 1F). Each cytosine base editor was co-transfected with UGI, gRNA G1 and gRNA G2. Among the tested base editors, MEGA-4 led the highest increase in GFP-negative cells (Figure 1F). Although AID-BE3 was also able to disrupt the GFP locus, it was significantly less efficient than MEGA-4. CP1012 CBEmax and BE4max did not show any activity in our assay.

### MEGA configurations impact the depth and breadth of genomic editing activity

**Figure 2.**
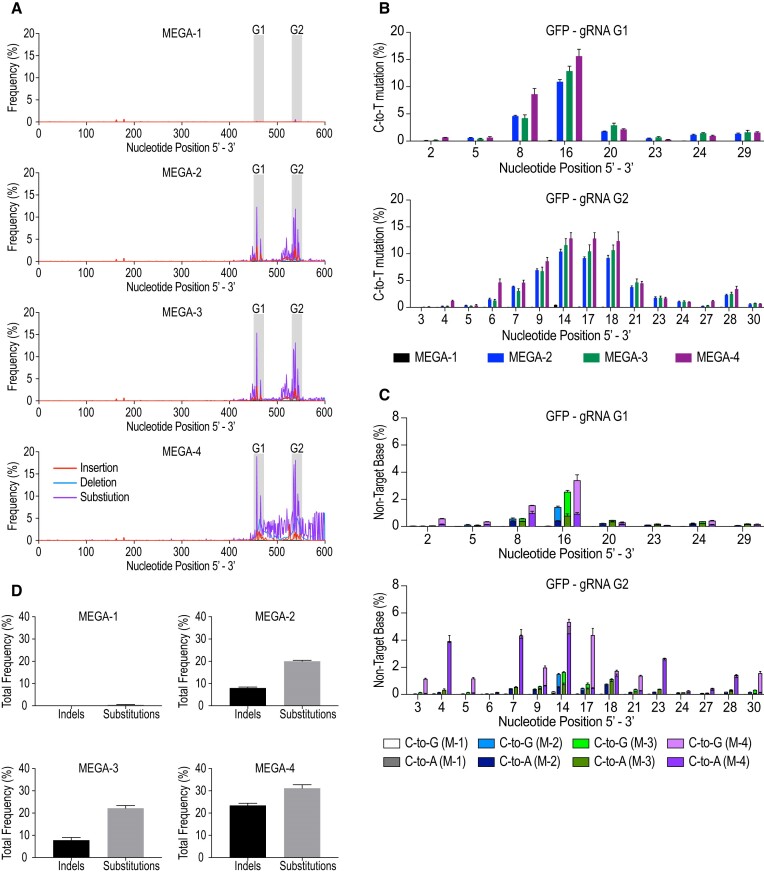
MEGA configuration impacts editing activity. (**A**) Sequencing histograms of the GFP amplicon visualise the editing outcome of each MEGA construct. Insertions, deletions and substitutions are shown in red, blue and purple, respectively. Protospacer regions are indicated with black errors and grey background. (**B**) Comparing targeted C-to-T editing frequency at *GFP* loci G1 and G2. Nucleotide numbering corresponds to their position relative to PAM sequence being at position 0. (**C**) Non-target base editing frequency is shown for each MEGA version at gRNA position G1 and G2. (**D**) Overall Indel and Substitution frequency for each MEGA configuration is shown. Experiments were done in triplicates and sequenced with a PacBio Sequel II machine. Mean with standard deviation of three independent experiments is shown. Three technical repeats were done per experiment. Statistical significance was calculated by a one-way ANOVA. (* *P* ≤ 0.5, ** *P* ≤ 0.001, *** *P* ≤ 0.0001 and **** *P* ≤ 0.00001).

Next, we performed deep sequencing to evaluate the editing efficiency of each MEGA construct together with UGI at both *GFP* loci G1 and G2. MEGA-1 did not show marked sequence alterations. Substitution mutations were, however, detected using MEGA-2, -3 and -4 (Figure 2A). They predominantly clustered around the corresponding gRNA targeting positions; yet, the 3 constructs displayed differential benefits. Notably, MEGA-2 and MEGA-3 editing was very comparable, further illustrating that transcriptional activation via VP64 does not significantly enhance AID activity. While MEGA-2 and -3 generate slightly lower editing frequencies, they display a more confined substitution spread than MEGA-4. MEGA-4 displayed higher peak mutations within the protospacer regions but also a wider spread of substitutions (Figure 2A). When looking at targeted C-to-T mutations at both loci, MEGA-2, -3 and -4 led to detectable editing at single base pair resolution (Figure 2B). Expectedly, MEGA-4 was the most potent editing construct followed by MEGA-3 and -2; with the mutations following a quasi-bell-shaped distribution with the highest frequency in the middle of the gRNA site. MEGA configurations also impacted base editing purity. MEGA-2 and MEGA-3 caused low levels of non-targeted base substitutions, but higher rates were observed in MEGA-4 (Figure 2C). Overall, MEGA-4 was the most effective construct with a total substitution frequency of 31.2 ± 1.54%; followed by MEGA-2 and -3 at 20.03 ± 0.40% and 22.2 ± 1.2%, respectively. MEGA-1 showed a meagre substitution frequency of 0.40 ± 0.14% (Figure 2D). Finally, we could also observe that the higher mutation frequency of MEGA-4 came at the expense of increasing Indels, too. MEGA-4 showed the highest Indel frequency of 23.44 ± 0.97%. In comparison, MEGA-2 and -3 had only minor Indel rates of 7.98 ± 0.45% and 7.81 ± 1.22%, respectively (Figure 2D).

### Deamination occurs preferentially at AID hotspots within the protospacer

**Figure 3.**
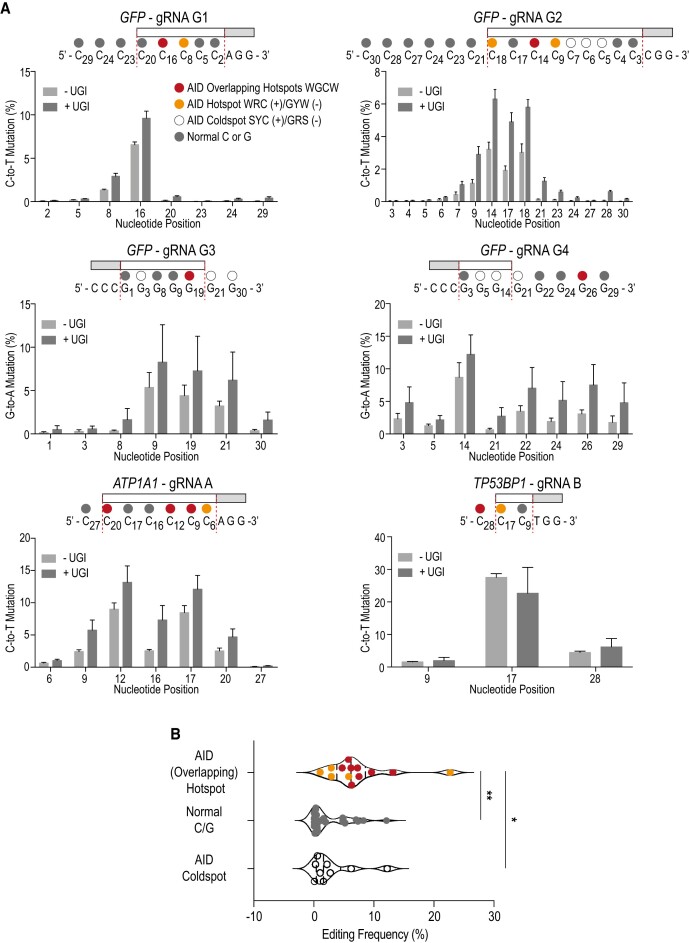
Deamination occurs preferentially but not only at AID hotspots within protospacer. (**A**) C/G-to-T/A mutation frequency of six different loci is compared with and without UGI co-expression. Reference sequence with gRNA protospacer region, PAM sequence and C’s/G’s are highlighted. Nucleotide numbering corresponds to their position relative to PAM sequence being at position 0. Mean with standard deviation of three independent experiments are shown. (**B**) Comparison of editing frequency depending on sequence context across all six tested sites. AID (overlapping) hotspots were compared to Cs/Gs without AID-related sequence context and AID coldspots. Mean with standard deviation of three independent experiments is shown. Three technical repeats were done per experiment. Statistical significance was calculated by a one-way ANOVA. (* *P* ≤ 0.5 and ** *P* ≤ 0.001).

**Figure 4.**
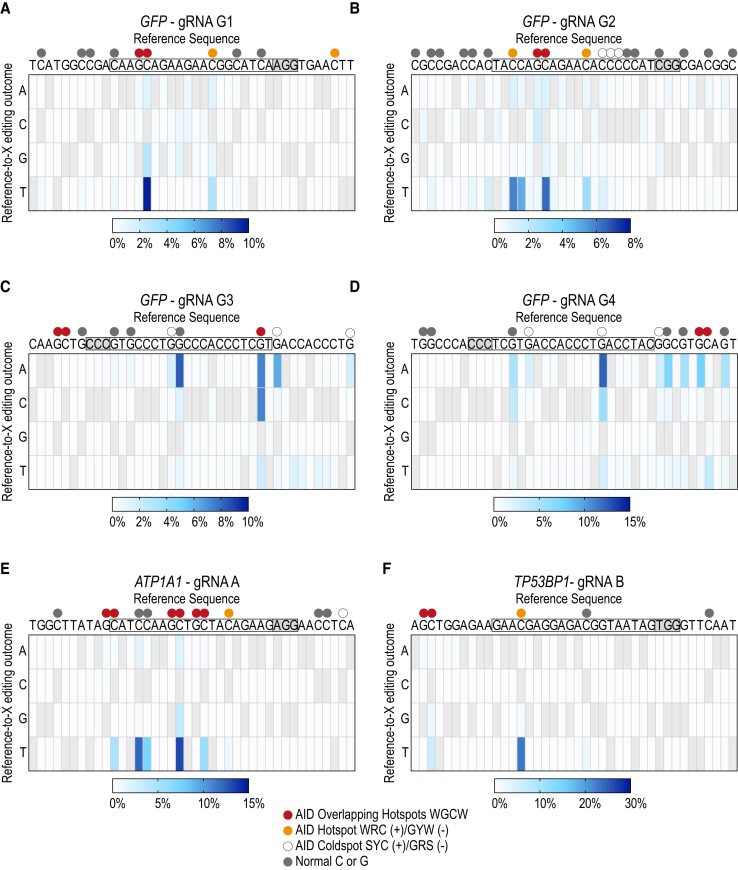
MEGA-4 shows high base editing diversity and Indel frequency. A-F) Editing of GFP locus G1 (**A**), GFP locus G2 (**B**), GFP locus G3 (**C**), GFP locus G4 (**D**), ATP1A1 locus A (**E**) and TP53BP1 locus B (**F**) with MEGA-4 and UGI. Heatmaps visualize the frequencies of all possible nucleotide substitutions at each position of the reference sequence. Reference bases were not considered and are greyed out. Mutations of G’s within the sense strand resulted from gRNAs targeting the antisense strand. gRNAs complementary to the antisense strand led to mutated C’s within the sense strand. Protospacer and PAM sequenced are highlighted. Coloured dots indicate specific sequence motifs within the quantification window. The mean of three independent experiments is shown. *GFP* loci G1–G4 were sequenced by PacBio single molecule long-read sequencing, while gene loci *ATP1A1* and *TP53BP1* were sequenced by Illumina Technology. A (adenosine), C (cytosine), G (guanosine), T (thymine).

To identify distinct mutation signatures of our MEGA system we mutated six different genomic loci in HEK293T-GFP cells with MEGA-4 and UGI. In addition to the four *GFP* loci we included two endogenous genes (Figures 3 and 4A-D). (i) Na^+^/K^+^ ATPase *ATP1A1* gene was targeted with a previously published gRNA herein referred to as gRNA A (29). (ii) For *TP53BP1* we designed gRNA B. All four *GFP* loci underwent single molecule long-read PacBio sequencing, while *ATP1A1* and *TP53BP1* were sequenced with Illumina technology. Both approaches gave comparable results (Supplementary Figure S4). C/G-to-T/A editing efficacy varied between the different loci. Again, most deamination events happened within the protospacer region where the base editor unwinds the DNA. For *GFP* loci G3 and G4 as well as for *TP53BP1*, however, C/G-to-T/A mutations also occurred beyond the protospacer (Figures 3A and 4C, D and F). Base editing efficacy was dependent on the target nucleotide position. MEGA-4 showed a broad editing window of approx. 20 nucleotides ranging from position nine to 29. Mutation events happened most efficiently between nucleotide positions 12 and 17 with the PAM sequence being at position 0 (Supplementary Figure S5). Also, we observed that MEGA-4 retained physiological AID sequence preferences. Hotspot and OHS motifs were preferentially deaminated across all tested sites (Figure 3B). For *TP53BP1* even the OHS outside the protospacer was also targeted (Figures 3A and 4F). In addition, MEGA-4 was able to mutate coldspot motifs at *GFP* loci G3 and G4 (Figures 3A and 4C and D). We also detected deamination of unrelated C’s/G’s that did not belong to specific motifs. Most often that was seen when they were close to AID hotspots. Especially, within the editing window AID hotspot and OHS showed the highest editing frequency (Supplementary Figure S5). This suggest that whilst the PAM position dictates the overall editing window, AID still displays WRC(Y) preference within that restricted window.

### MEGA-4 shows high base editing diversity and Indel frequency

Overall, C/G-to-T/A substitutions were the most frequent types of edits seen with MEGA-4 (Figures 3A and 4). Co-expressing UGI did enhance mutagenesis at most targetable sites (Figure 3A). Especially, editing outside the protospacer region improved. We could also detect C-to-G/A or G-to-C/T mutations (Figure 4 and Supplementary Figure S6). Depending on the targeted loci UGI influenced base editing purity differently. For gene loci *ATP1A1* and *TP53BP1* a decrease in non-specific C/G-to-T/A substitutions were observed when UGI was added (Supplementary Figure S6). However, no decrease in non-targeted base editing was seen at all four *GFP* loci. While base editing purity did not change for locus G3, induction of non-targeted substitutions increased at GFP position G1, G2 and G4. Again, MEGA-4 did not only lead to target base substitutions but also to a high Indel frequency (Supplementary Figure S7). UGI helped to decrease Indel frequency at *ATP1A1* and *TP53BP1* but not at the other four *GFP* loci. Instead, it led to a slight increase of Indels.

### MEGA can mimic SHM and CSR by inducing mutations at a refractory ig gene

**Figure 5.**
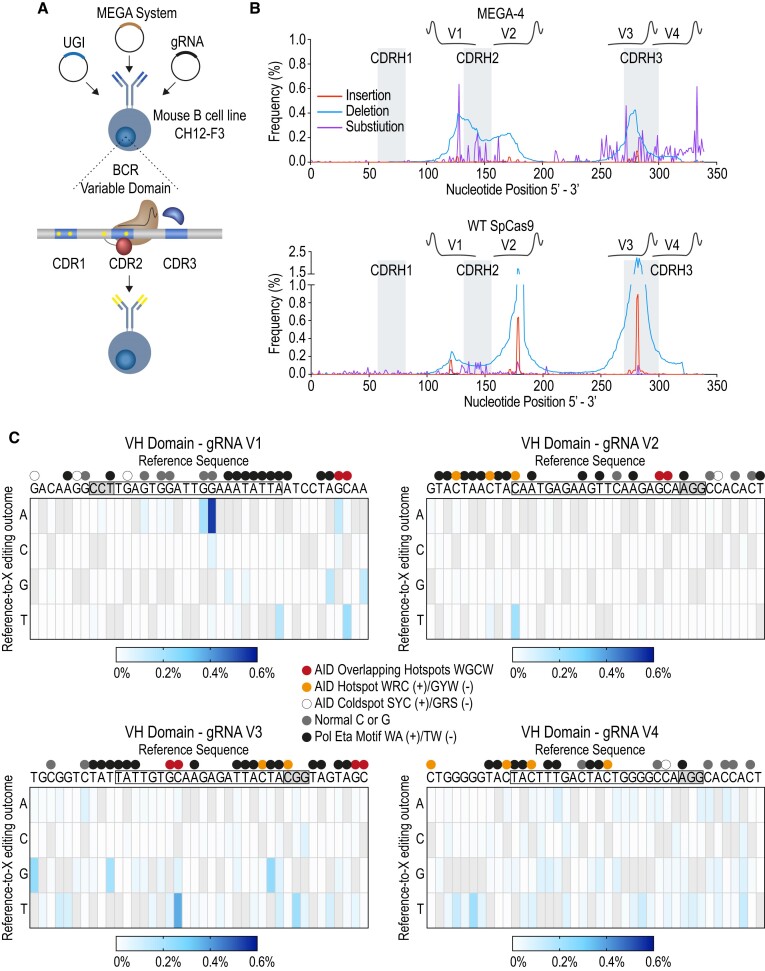
MEGA-4 induces broad mutation pattern in murine variable heavy chain domain. (**A**) Individual plasmids encoding for MEGA-4, UGI and four different CDR-targeting gRNAs were electroporated into the murine B cell line CH12-F3. (**B**) Sequencing histogram of the variable heavy chain domain. Insertions, deletions, and substitutions are represented in red, blue and purple, respectively. (Upper Panel) MEGA-4 together with UGI and four gRNAs. (Lower Panel) WT *S. pyogenes* Cas9 with four gRNAs. CDR1–3 as well as the protospacer regions of each gRNA are highlighted. (**C**) Heatmaps represent targeted single base mutations around gRNA position V1, V2, V3 and V4. Reference sequences with highlighted protospacer region and PAM sequence are given for each locus. Coloured dots indicate specific sequence motifs within the quantification window. Three independent experiments were sequenced using Illumina technology. BCR (B Cell Receptor), CDR (Complementarity Determining Region), Pol Eta (polymerase eta), WT SpCas9 (wild type *S. pyogenes* Cas9).

**Figure 6.**
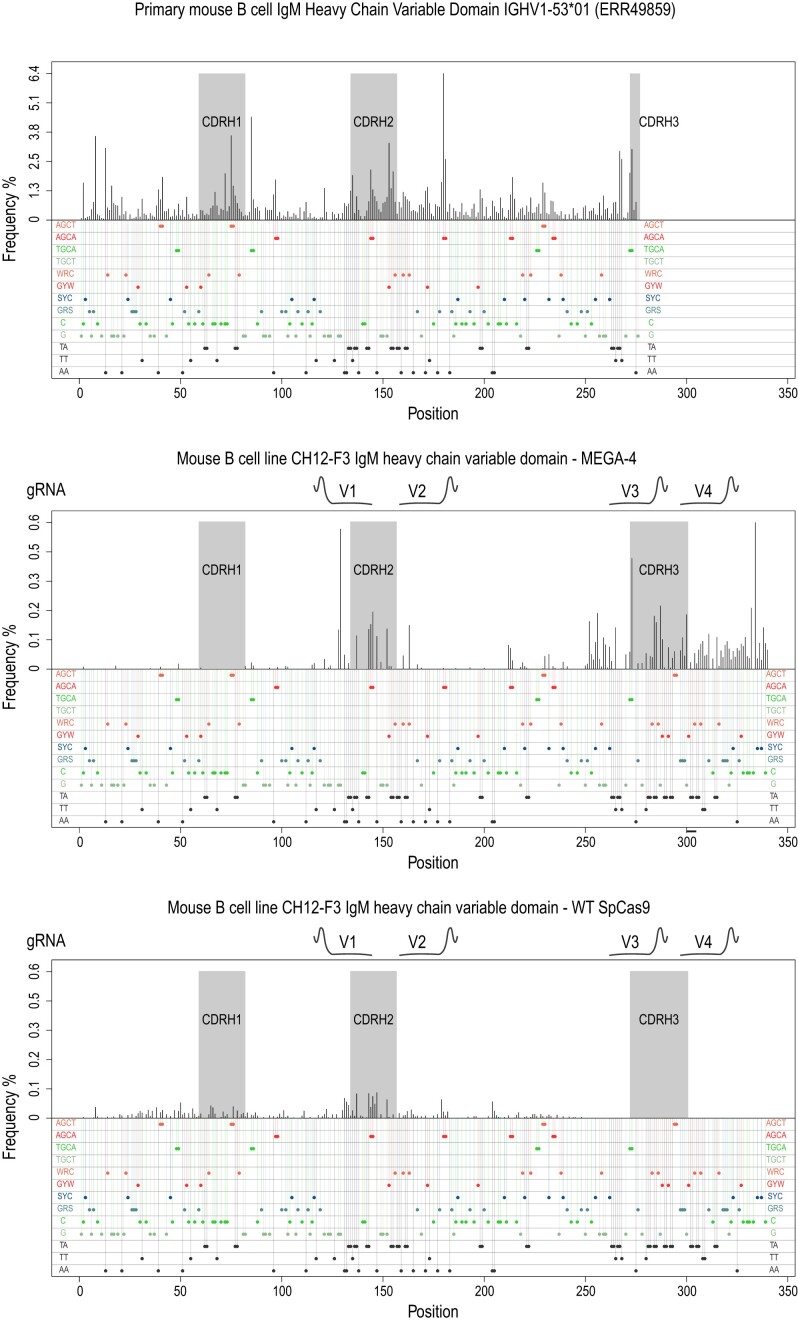
Comparing primary mouse B cell somatic hypermutation with MEGA-4 and wild type Cas9 induced mutation spectrum. CH12-F3 cells mutated with MEGA-4 show similar mutation spectrum at the respective gRNA positions as primary mouse B cells. Wild type Cas9 only showed low level of substitutions. Some of which were gRNA independent. For comparison with primary cells, mouse B cells with the same germline sequence as the CH12-F3 were used.

The murine IgM-positive B cell line CH12-F3 is believed to be refractory for SHM whilst mediating CSR at high frequency following cytokine induction (7). Considering our MEGA architecture retained AID’s mode-of-action, we chose to use it as a proof-of-concept for SHM mimicry. CH12-F3 cells were electroporated with MEGA-4 together with UGI and four CDR-targeting gRNAs (V1-4) in both leading and lagging strands (Figure 5A). Subsequently, the variable heavy chain domain was sequenced by Illumina technology. At the four gRNA locations we succeeded in creating significant single base substitutions (Figure 5B, magenta). Low levels of localized deletions (Figure 5B, blue) were also detectable at the respective sites with little or no insertions (Figure 5B, red). MEGA-4 was able to induce targeted single base substitutions mutations while wild type Cas9 only created overlapping Indels (Figure 5B). Base editing predominantly happened at AID hotspots (Figure 5C). For locus V1 we observed A-to-G mutations at 5′ WA 3′ motifs indicating the involvement of error-prone polymerase eta. For gRNA loci V3 and V4 the mutation pattern was scattered and did not follow defined sequence motifs (Figure 5C). Using the mutation analysis tool SHMprep, we were able to compare our data to the physiological mutation spectrum of a primary mouse B cell with the same germline sequence (Figure 6) (33). At the respective gRNA positions, MEGA-4 induced mutations resembled physiological SHM. Wild type Cas9 did not create a comparable mutation spectrum. CSR represents the second AID-driven event during antibody maturation. AID-mediated deamination activity at both strands of immunoglobulin switch regions eventually lead to DNA DSBs and the change in antibody isotype (36). When further analysing the deletion sites we detected the occurrence of short microhomologies (Supplementary Figure S8A), which are by-products of DNA repair, especially, during CSR. Whilst MEGA-4 microhomologies were mostly between 1 and 2 nucleotides long, wild-type Cas9 showed predominantly microhomologies with 3 nucleotides in length. To further compare microhomology size, we took advantage of a recent study we conducted on exonuclease 1 (Exo1) (37), which is a pleiotropic DNA repair factor. We could show that MEGA-4 displays comparable microhomology patterns as our wild-type Exo1 mouse model but different than null or nuclease-defective Exo1 mice (Supplementary Figure S8B).

### MEGA-1 displays low genomic but high epigenomic editing through active cytosine demethylation

**Figure 7.**
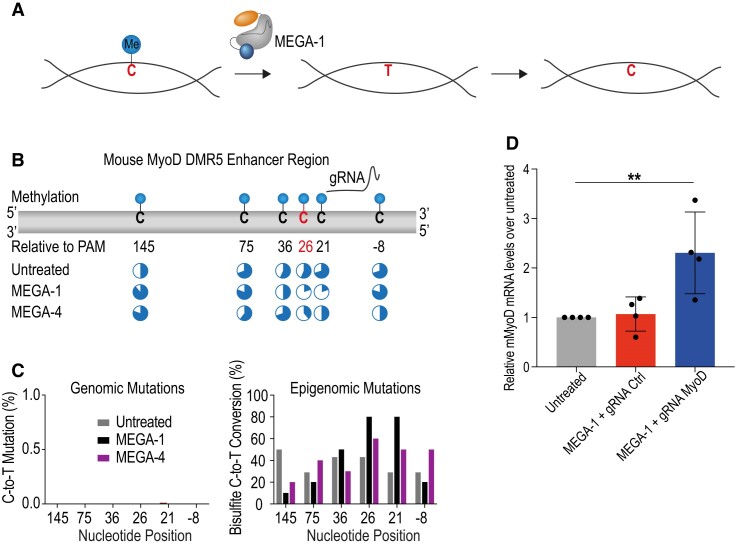
MEGA-1 has low mutagenic but high epigenetic activity. (**A**) MEGA enables targeted demethylation of 5mC’s. Deaminated 5mC’s are recognized as T’s and will be replaced enzymatically with non-methylated C’s. (**B**) Schematic representation of targeted mouse MyoD enhancer region. The methylated AID hotspot is highlighted in red. Localisation of the MyoD gRNA is depicted. Pie charts underneath indicate the percentage of clones with methylated (blue slices) or unmethylated (empty slices) C’s. Nucleotide numbering refers to the PAM sequence being at position 0. (**C**) (Left panel) Frequency of genomic C-to-T mutations. (Right panel) Frequency of epigenomic demethylation. In total, 6–10 individual clones were analysed. (**D**) Relative MyoD gene expression normalized to housekeeping gene *Actin B* and then again normalized to non-transfected cells. MyoD gene expression was analysed 48 h post-transfection by RT qPCR. Each dot represents an independent experiment. Statistical significance was calculated by a one-way ANOVA and multiple comparison (** *P* ≤ 0.001). 5mC (5-methylcytosine), RT qPCR (real time quantitative PCR).

Besides genomic editing AID-dependent deamination leads to 5mC demethylation (17). Targeted 5mC deamination results in a T–G mismatch, which resolves by replacing the T through an unmethylated C (Figure 7A). Despite its low genomic editing activity, we wanted to test whether MEGA-1 had instead epigenomic editing potential. To do this, we targeted the MyoD DMR5 enhancer region of the murine 3T3 fibroblast cell line with our MEGA system. MyoD is a well-defined master transcriptional regulator for muscle cell development (38). We used a previously published MyoD-specific gRNA which bound adjacent to a methylated AID hotspot (30). Potential epigenomic changes as well as genomic changes were detected by bisulfite and Illumina sequencing, respectively (Supplementary Figure S9A). In total six 5mCs were near the gRNA (Figure 7B). Compared to untreated non-transfected cells MEGA-1 led to efficient demethylation of the three 5mCs at position 21, 26 and 36 (Figure 7B and C and Supplementary Figure S9B). Weak demethylation activity was also seen with MEGA-4, but MEGA-1 was more efficient in editing position 21 and 26 (Figure 7C and Supplementary Figure S9B). At the methylated AID hotspot at position C_26_ and the adjacent 5mC at position C_21_ MEGA-1 demethylated 80% of all bisulfite-treated clones (Figure 7C and Supplementary Figure S9B). Bulk deep sequencing with Illumina technology confirmed that the bisulfite-sequencing results did not result from genomic 5mC-to-T mutations (Supplementary Figure S9A). Neither MEGA-1 nor MEGA-4 were able to mutate 5mC on a genomic level (Figure 7C). MyoD is expressed only during muscle development. Its expression is tightly regulated through the methylation status of its enhancer region (30,38). The methylation status of the AID hotspot, we targeted, was particularly shown to be critical for transcription (38). We asked if the demethylation activity of MEGA-1 was sufficient to induce MyoD expression in our murine fibroblast cell line. To that end, we detected a 2.3 ± 0.83-fold increase in MyoD expression compared to untreated control when transfecting with MEGA-1 and gRNA MyoD (Figure 7D). When using another published gRNA (gRNA Ctrl) that binds approx. 170 nucleotides upstream of gRNA MyoD we did not detect a significant increase in expression (Figure 7D). In addition to the MyoD enhancer region, we targeted the promotor region of the oxytocin receptor (OxyR). Like MyoD, the oxytocin receptor locus is silenced in mouse fibroblast cell lines. Two critical transcription factor binding sites, namely for estrogen and SP-1, within the oxytocin receptor promotor region have been identified. The methylation status of these sites dictates the expression level the oxytocin receptor (39). To induce gene expression by MEGA-dependent demethylation we designed gRNA OxyR1 and gRNA OxyR2, which targeted the estrogen and SP-1 binding site, respectively (Supplementary Figure S10A). We observed that only MEGA-4 with gRNA OxyR2 led to a significant induction in oxytocin receptor gene expression (Supplementary Figure S10B). No change in gene expression was observed with gRNA OxyR1. MEGA-1 was not able to induce oxytocin receptor gene expression neither with gRNA OxyR1 nor OxyR2.

## Discussion

In this work, we re-capitulate for the first time the full functional spectrum of human AID activity *ex vivo*. Our novel MEGA editing system allowed targeted SHM-like single base editing, CSR-like DSB induction, and 5mC DNA demethylation. In brief, protein architecture and configuration strongly influenced genetic and epigenetic editing.

### Full-length human AID and the transcriptional activator VP64 do not synergise

Studies with non-B cell lines have shown that ectopic expression of full-length wild type AID is able to induce SHM-like events. Such approaches suffered from poor mutation rates and happened randomly (40,41). The development of CBEs opened new possibilities for *ex vivo* genome editing with human AID. However, previously published base editors which linked full-length wild type AID to dCas9 lacked editing activity. One possible reason could be a limited ssDNA substrate accessibility during Cas9-dependent R-loop formation. *In vivo* two mechanisms are proposed to produce ssDNA for AID. During gene transcription stalling of RNA polymerase II and its co-factor Spt5 causes a premature transcription termination and subsequently ssDNA exposure (42,43). Alternatively, the collision between a transcription bubble and a replication fork can also lead to prolonged substrate presentation (44). We postulated that AID’s *ex vivo* activity would improve over a more physiological substrate. Therefore, a transcription bubble was created by fusing the potent minimal transcription activator VP64 to MEGA-1. Studies have shown that linking VP64 to dCas9 can induce targeted gene transcription (45,46). When testing MEGA-1 we could not detect any editing effect neither phenotypically through GFP fluorescence loss nor at the genomic level. It remains possible that either additional substrate conformations are needed and/or auxiliary AID factors are required to induce wild type AID activity. Potentially to protect the genome from catastrophic events (47). And these regulatory factors may not be reconstituted in our setting. Interestingly, AID-BE3 uses human wild type AID and showed activity in our GFP disruption assay. Most likely, the interplay with nCas9 helps to enhance its mutagenic ability (48).

### MEGA configurations impact the performance of human AID

Distinct point mutations and truncations of human AID help to overcome the suboptimal editing activity *ex vivo* (22,25,26,49). Indeed, when replacing the full-length human AID with the engineered hyperactive human AID*Δ all MEGA variants showed efficient editing. Compared to full-length protein AID*Δ lacks the C-terminal nuclear export signal (NES) and harbours three amino acid substitutions (23). Both modifications have been correlated with enhanced mutation frequency (50,51). Interestingly, through the different configurations we could fine tune the editing activity of our MEGA system. Among the three AID*Δ variants MEGA-3 had an intermediate activity considering GFP disruption, targeted C-to-T editing and base editing purity. MEGA-2 containing VP64 performed least among the three constructs in the GFP disruption assay. Compared to MEGA-3, however, it only showed a slight decrease in the overall substitution frequency. In addition, it had the best base editing purity profile. The recruitment of the transcription machinery potentially caused steric hinderances at the genomic target site. With the effect of less overall editing but with more stringent tendency to C-to-T mutations. In the context of glycosylase cytosine-to-guanine base editors, addition of VP64 to the N-terminus showed a significant improvement in editing (52). We positioned VP64 to the C-terminus, which could explain why we did not observe any effect. However, it needs to be considered that glycosylase base editors follow a different mutagenesis mechanism than CBEs. MEGA-4, on the other hand, was the most potent but also the most diverse base editor in our system. Exchanging dCas9 for nCas9 had, indeed, a strong effect on the global mutagenic potential of AID. Like AID under physiological conditions MEGA-4 did not only induce C/G-to-T/A mutations but also led to other types of base substitutions. Hence, offering the possibility for enhanced sequence diversification. We did not observe a marked strand bias for MEGA-4 in GFP disruption, when comparing the two opposite gRNAs, G1 and G1’. This was in line with previous work, that did not show a strong strand bias for AID under physiological conditions (53). However, targeting the negative strand and adding UGI resulted in less GFP loss. For gRNA G1, which targets the positive strand, our analysis showed a higher C-to-T editing at position 16 when adding UGI. Ultimately, causing a higher frequency of premature stop codons, which translated in a higher level of GFP loss. UGI is known to enhance substitutions and reduce Indel formation by preventing uracil DNA glycosylase to form abasic sites (20,48). It is possible that GFP loss with gRNA G1’ was predominantly caused by Indel formation since gRNA G1’ did not overlap with any premature stop codon positions. Hence, UGI may not enhance but rather negatively affect the gRNA G1’outcome on GFP loss.

### The interplay between AID and nCas9 mimics CSR induction

Besides expected single base substitutions, we observed a high Indel frequency with MEGA-4 (Supplementary Figure S7). Standard CBEs are reported to catalyse low DSB frequency which eventually result in Indels (54). The phenotypic GFP loss caused by MEGA-4 was most likely not exclusively the result of targeted C/G-to-T/A mutations; sequence frameshifts may have also contributed to the detected phenotype. High Indel rates have been, however, also reported for similar base editors which combine hyperactive forms of human AID and nCas9 (25,49). It is well known that during CSR, AID is capable of inducing DSBs (55).The Ig switch regions contains a higher density of AID OHS, which – only in part – explains the difference between SHM and CSR (56). While SHM can happen independent of secondary DNA structures; CSR efficacy is highly dependent on them. The high G-cluster density in the switch regions facilitate R-loop as well as G4 structure formation (57,58). It is proposed that they slowdown RNA polymerase II processivity which ultimately causes prolonged substrate exposures (56,57,59). Furthermore, a structural study demonstrated that G4 structures have a higher affinity towards AID and promote its oligomerisation *in vitro* (58). Eventually, the structural features allow a high density of mutations to happen on both DNA strands where individual single strand breaks will accumulate to DNA DSBs. With MEGA-4 we were able to recapitulate the requirements for single point mutations to be processed to DSBs. Whether or not we were able to induce similar DNA structures as in CSR remains to be elucidated. Previous work has shown that wild type Cas9 can induce CSR in B cells ex vivo (60,61). The presence of microhomologies at the deletion sites indicate similarities to physiological CSR (37). Most likely the combination of nicking the non-edited strand by nCas9 and creating an abasic site following C deamination caused the disruption of the target locus. That the combination of hyperactive AID and nCas9 is needed to increase DSB formation could be highlighted when observed in context of our dCas9 base editors MEGA-2 and -3. Even though they both generated Indels, they occurred with about three-times lower frequency than with MEGA-4. Moreover, base editors which link wild type full-length AID with nCas9 do not induce Indel frequencies as high as we see (48).

### The MEGA system induces SHM *ex vivo*

A major advantage of AID base editors is their ability to edit C’s within 5′-GC-3′-contexts as part of the WRC consensus preferred by AID proteins. APOBEC base editors instead favour 5′-TC-3′ sequences (25,49). Hence, base editors can reflect the endogenous sequence preferences of the deaminase used. Upon binding AID slides and jumps along the ssDNA to search for hotspot motifs (62). Our MEGA system clearly showed a comparable mode-of-action. If AID hotspot and OHS motifs were present, they were preferentially targeted over non-hotspots. Coldspots and unrelated C’s which would be omitted or less likely targeted by physiological AID were found to be mutated as well. The overall nucleotide sequence preference seemed to be less restricted with hyperactive AID*Δ and resulted most likely from the interplay between AID hotspot and nucleotide position as it defines the editing window. Inducing SHM-like mutations in the Ig locus of the murine B cell line CH12-F3 further proofed how close we mimicked AID *ex vivo*. To our knowledge we are the first who could successfully diversify an endogenous Ig variable domain with a CBE. Besides C/G-to-T/A mutations we detected A-to-G substitutions. As they fall into 5′-WA-3′ motifs we concluded the low-fidelity DNA polymerase eta to be very likely responsible for such modifications, akin to its role in SHM physiologically. This is of particular interest as error-prone DNA repair represents the second phase of SHM (63). In addition, we induced different levels of Indels within the variable domain (Figure 5B). During physiological SHM this happens as well to further broaden the Ig repertoire, often creating flexibility to the antibody binding affinity (64,65).

### MEGA promotes AID’s epigenetic function

Epigenomic editing has attracted a lot of attention over the recent years. Through targeted modifications of the promotor and enhancer methylome a transient regulation of gene expression can be achieved (66). *In vivo* active 5mC demethylation is mainly catalysed by members of the ten-eleven translocation methylcytosine dioxygenase (TET) enzyme family. Linkage of TET enzymes with dCas9 represent the most widely used programmable epigenomic editors so far (30,66). While oxidation of 5mC through TET creates 5-hydroxymethylcytosine (5hmC) and other derivatives thereof, AID-dependent deamination changes 5mC directly to a T (14,67). Targeting a highly methylated region of the MyoD enhancer region impressively proved that our modular MEGA system with full-length human AID can efficiently demethylate specific 5mC’s. In a recently published work, the same region of MyoD was edited with dCas9-TET1 but together with four gRNAs spanning a region over 200 nucleotides (30). By only using one of these four gRNAs MEGA-1 achieved a comparable increase in MyoD expression as reported with dCas9-TET1. While TET-fusion constructs have a broad and unspecific demethylation activity, our MEGA system was able to edit the essential reported 5mC site in a narrow editing window of 36 nucleotides to induce gene expression (38). The absence of genomic 5mC-to-T mutations with MEGA-1 confirmed that the deamination activity exclusively affected the epigenome but not the genome. Interestingly, MEGA-4 did not have any mutagenic activity, even though it is the variant with the strongest deamination phenotype. However, highly methylated genomic regions represent a challenging target for base editors in general (68). We expanded our system to the oxytocin receptor promotor region, another silenced gene in fibroblast cell lines (39). Unlike for MyoD, MEGA-4 but not MEGA-1 was able to induce gene expression. A potential explanation why MEGA-1 did not show an effect could be the position of the respective methylated CpG sites. MEGA-4 could have been more efficient in demethylating the 5mC within the protospacer than MEGA-1. Due to limited PAM availability, we were not able to position gRNA OxyR1 in close proximity to the estrogen binding site. Hence, we potentially did not reach the critical 5mC, although its methylation status was reported to correlate with oxytocin receptor gene expression (39). Eventually, our system could be useful to understand the role of specific CpG clusters through its precise demethylation window at the single base de-methylation resolution which has never been achieved before. Whether or not these epigenomic changes are permanent or transient remains to be determined. It is possible that DNA methyl-transferases can counteract our epigenomic editing. That is why, future experiments could benefit from knockdown of key DNA methyl-transferases and/or the use of chemicals that can neutralize their function (30).

### Future perspective

For the first time we demonstrated that the full activity spectrum of AID can be translated *ex vivo*. Current genome or epigenome editors represent specialised molecular tools with single functions. The MEGA system, however, is a true multifunctional ‘genomic swiss army knife’. To our knowledge, there is no other molecular tool that bundles single point mutations, DSBs and 5mC demethylation all in one system. Depending on the configuration of the system one function can be favoured over the other. We are optimistic that our tool will help to improve our understanding of AID’s basic biology as well as the DNA methylome. Further we think our system will enhance protein engineering and diversification, such as ex vivo antibody engineering.

## Supplementary Material

gkad1118_Supplemental_File

## Data Availability

Sequencing data are available through GEO accession GSE206445. Mutation analysis scripts are in Github (https://gitlab.com/maccarthyslab/SHMPrep; https://gitlab.com/maccarthyslab/SHMServer).
