# Supplementary material for: Modular cytosine base editing promotes epigenomic and genomic modifications
Source: Nucleic Acids Res. 2023 Nov 22;52(2):e8. doi: 10.1093/nar/gkad1118 (PMC10810192; doi:10.1093/nar/gkad1118)
Supplement: gkad1118_Supplemental_File [file gkad1118_supplemental_file.pdf]

Modular Cytosine Base Editing Promotes Epigenomic and Genomic Modifications

Julian Weischedel[1*], Laurence Higgins[2*], Sally Rogers[2], Anna Gramalla-Schmitz[1], Paulina Wyrzykowska[1], Simone Borgoni[1], Thomas MacCarthy[3], and Richard Chahwan[1#]

[1] Institute of Experimental Immunology, University of Zurich, Zurich 8057, Switzerland

[2] Current address: Living Systems Institute, University of Exeter, EX4 4QD, UK

[3] Department of Applied Mathematics & Statistics, Stony Brook University, NY 11794-3600, USA

Supplementary Data

- **Supplementary Figures S1 – S10**
- **Supplementary Tables S1 – S4**

* Authors contributed equally to the work

# Correspondence author: chahwan@immunology.uzh.ch (Tel: +41 446353710)

Keywords: genome editing, epigenome editing, CRISPR, AID, double strand break, SHM

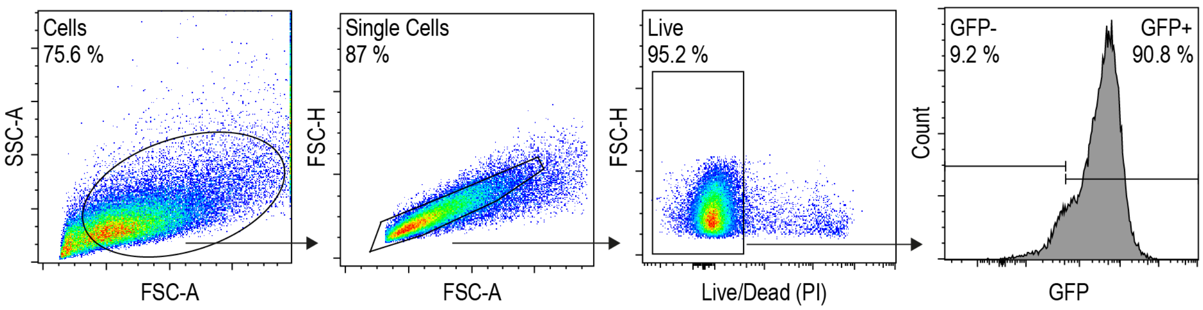

Figure S1: Gating strategy GFP disruption assay. Cells were initially gated based on morphology. Subsequently duplets and dead (PI-positive) cells were excluded. Remaining events were considered for analysis.

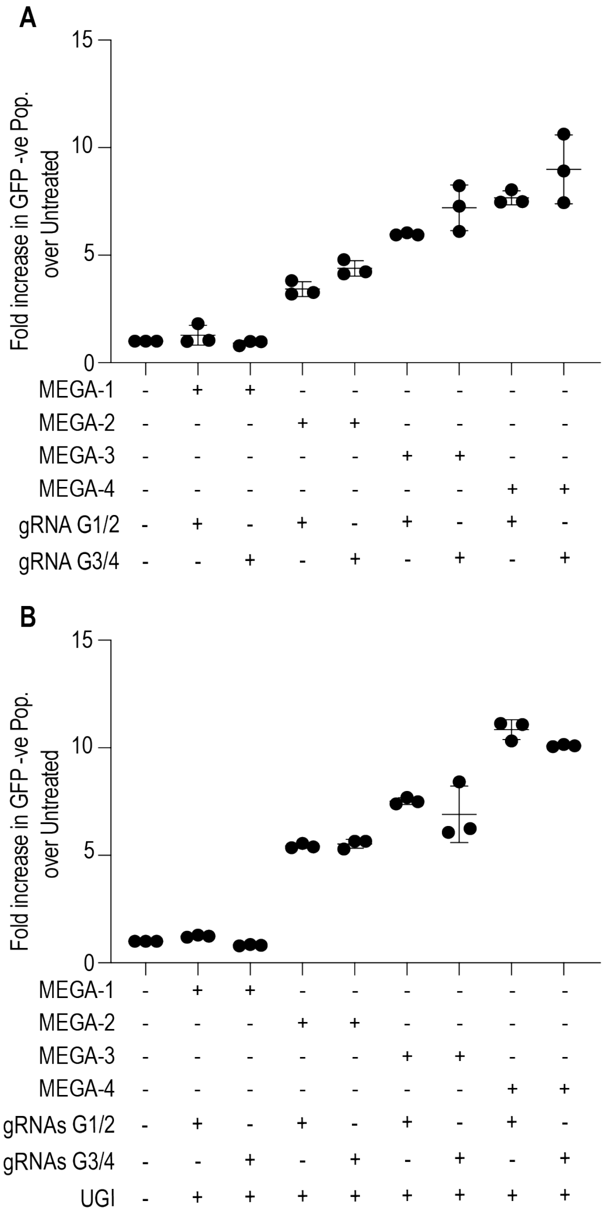

Figure S2: MEGA Base Editors do not show strand bias in GFP disruption assay. A-B) No significant difference in MEGA-1, -2, -3 or -4 dependent GFP loss when using gRNA combination G1 and 2 or G3 and 4. Experiments were done without UGI (A) or with UGI (B). All results are normalized and shown as fold increase of GFP-negative population over non-transfected control HEK293T-GFP cells. Mean with standard deviation of three independent experiments is shown. Three technical repeats were done per experiment. Statistical significance was calculated by a one-way ANOVA.

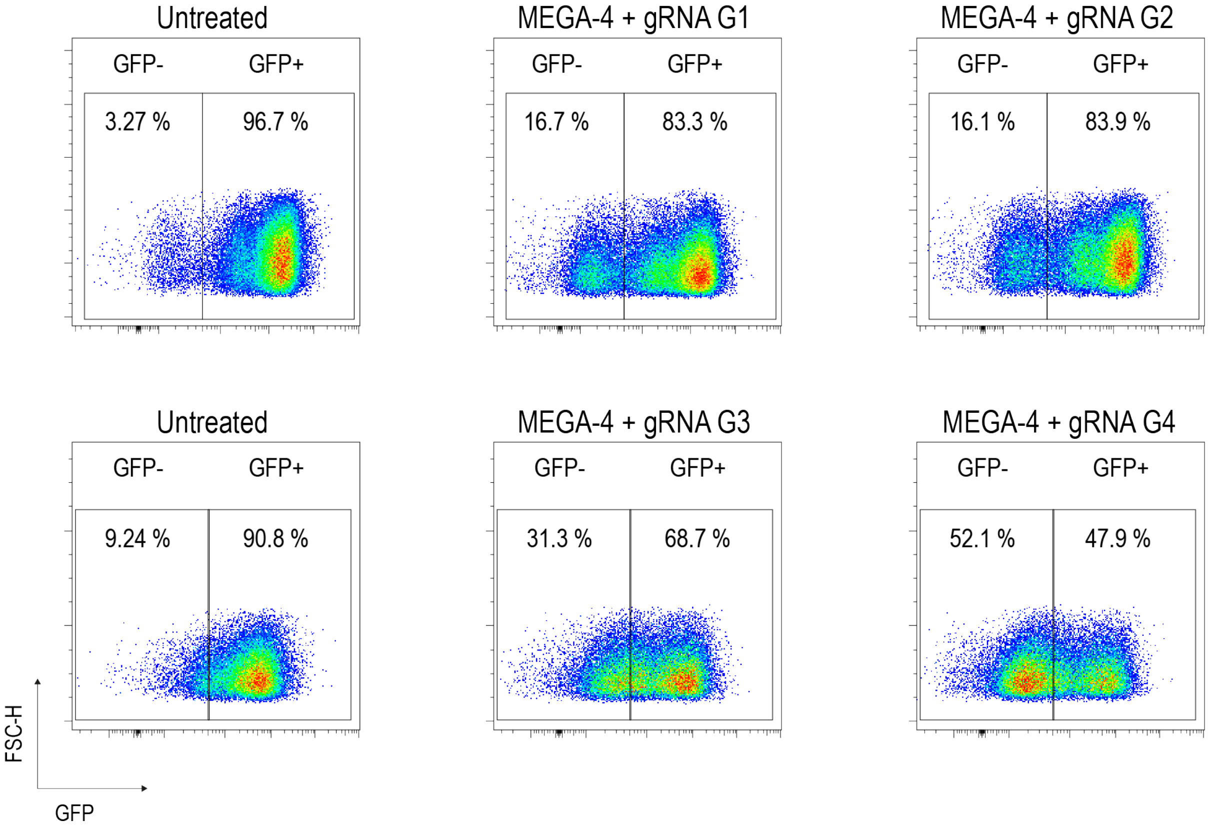

Figure S3: Targeted GFP disruption with MEGA-4 Base Editor. Representative FACS blots show the distribution of GFP-negative and -positive cells depending on the indicated condition. Changes in GFP-negative cell population of transfected HEK293T-GFP cells were normalised to GFP-negative population of non-transfected HEK293T-GFP cells.

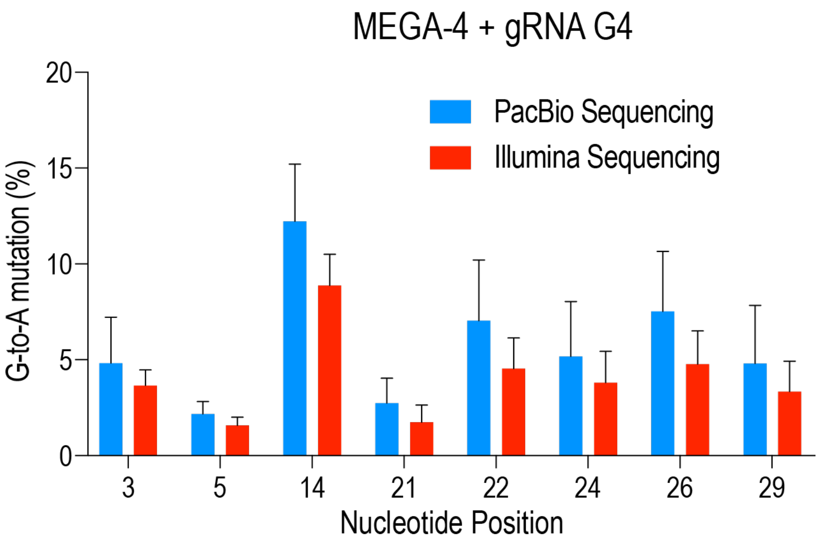

Figure S4: Comparison PacBio vs. Illumina Sequencing. Comparison between PacBio single molecule long-read sequencing and Illumina sequencing of the GFP locus G4. Nucleotide numbering corresponds to their position relative to PAM sequence being at position 0.

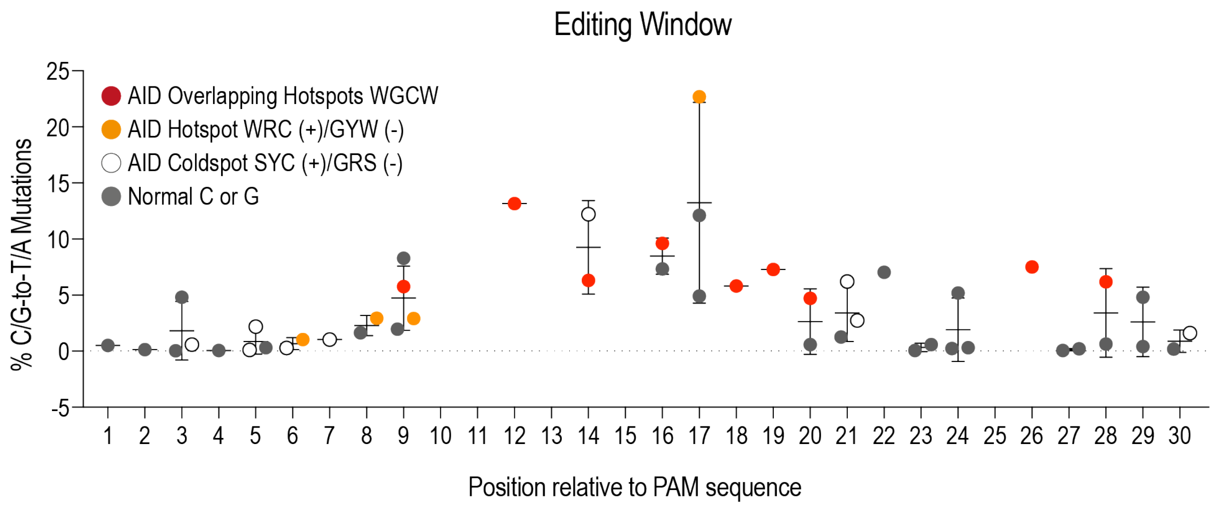

Figure S5: MEGA editing window. In relation to the PAM sequence at position 0 mutations happened predominantly but were not limited to the second half of the protospacer region. The target window had an approximately size of 20 nucleotides. Mutation frequency peaked between position 14 and 17. Nucleotide numbering corresponds to their position relative to PAM sequence being at position 0. Mean with standard deviation of three independent experiments is shown.

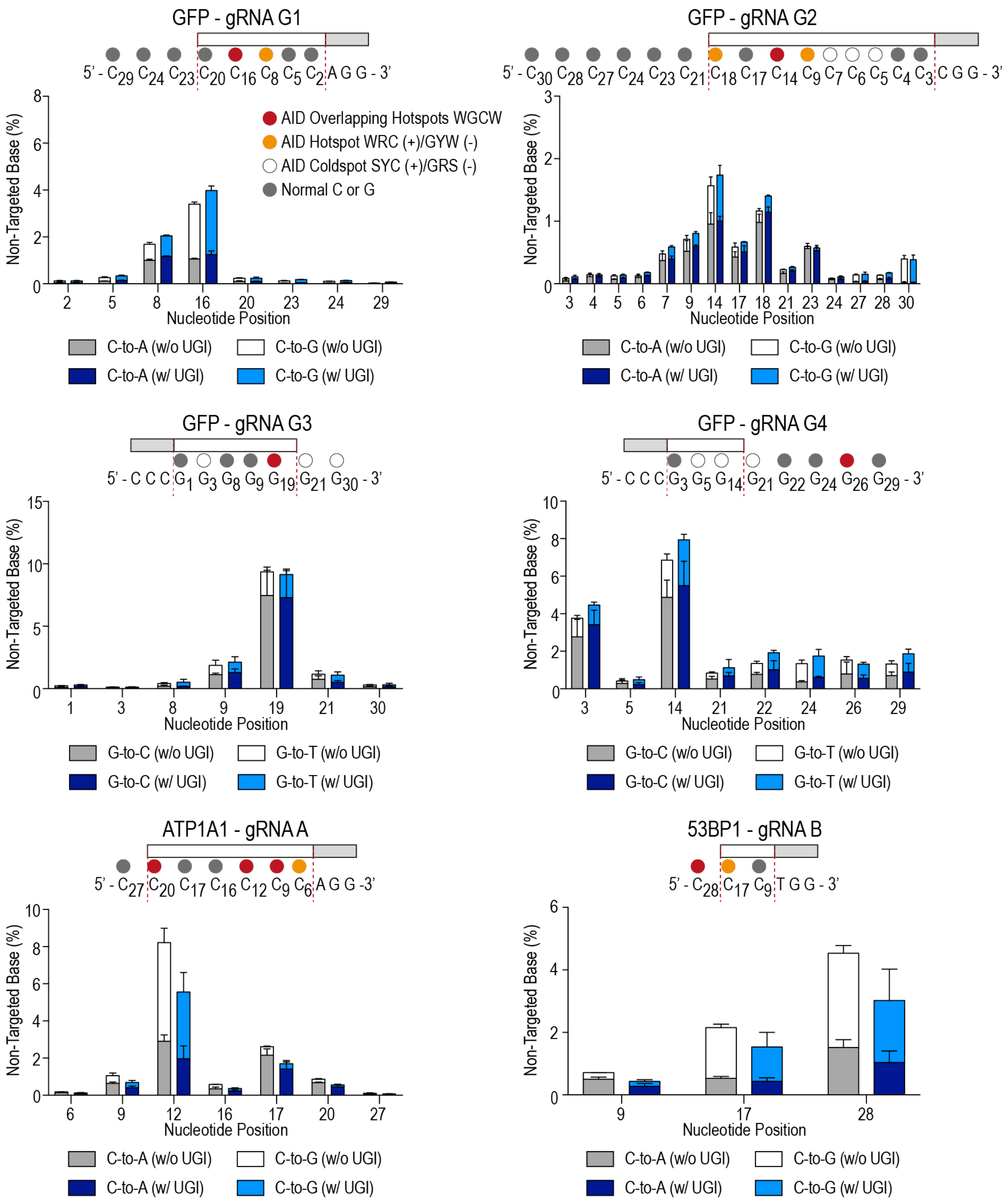

Figure S6: Diverse base editing outcome of MEGA-4. C-to-A/G and G-to-C/T mutation frequency is shown for six loci, respectively. Base editing purity with and without UGI is compared. For each locus the respective reference sequence with the protospacer region, PAM sequence, and C's/G's are highlighted. Coloured dots indicate specific sequence motifs

within the quantification window. Nucleotide numbering corresponds to their position relative to PAM sequence being at position 0. Mean with standard deviation of three independent experiments is shown. *GFP* loci G1 – G4 were sequenced by PacBio single molecule long-read sequencing, while gene loci *ATP1A1* and *TP53BP1* were sequenced by Illumina Technology.

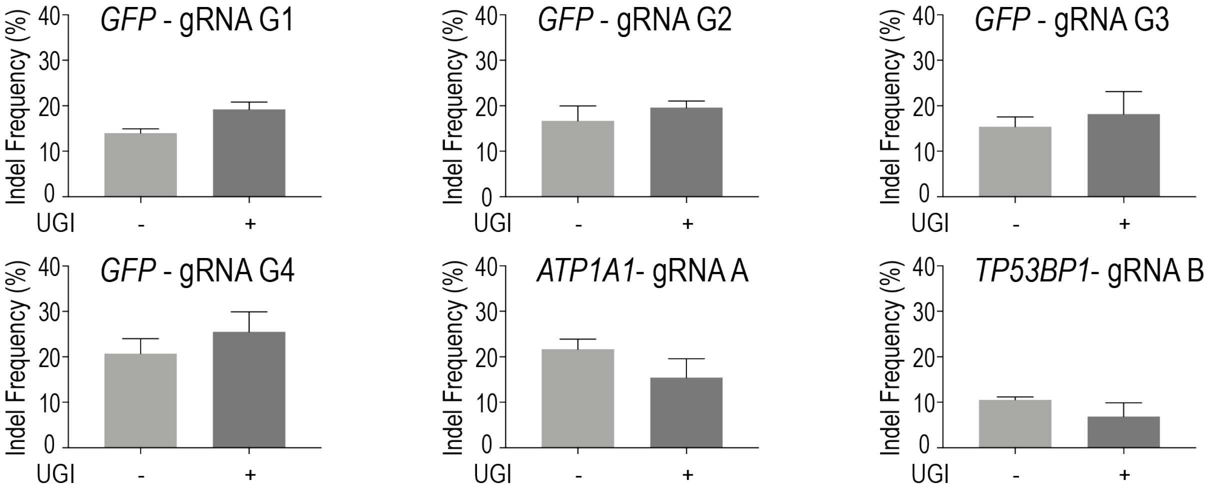

Figure S7: Impact of UGI on MEGA-4 Induced Indel Frequency. Total Indel frequency with and without UGI was calculated for each locus. Mean with standard deviation of three independent experiments are shown. *GFP* loci G1 – G4 were sequenced by PacBio single molecule long-read sequencing, while gene loci *ATP1A1* and *TP53BP1* were sequenced by Illumina Technology. Mean with standard deviation of three independent experiments is shown.

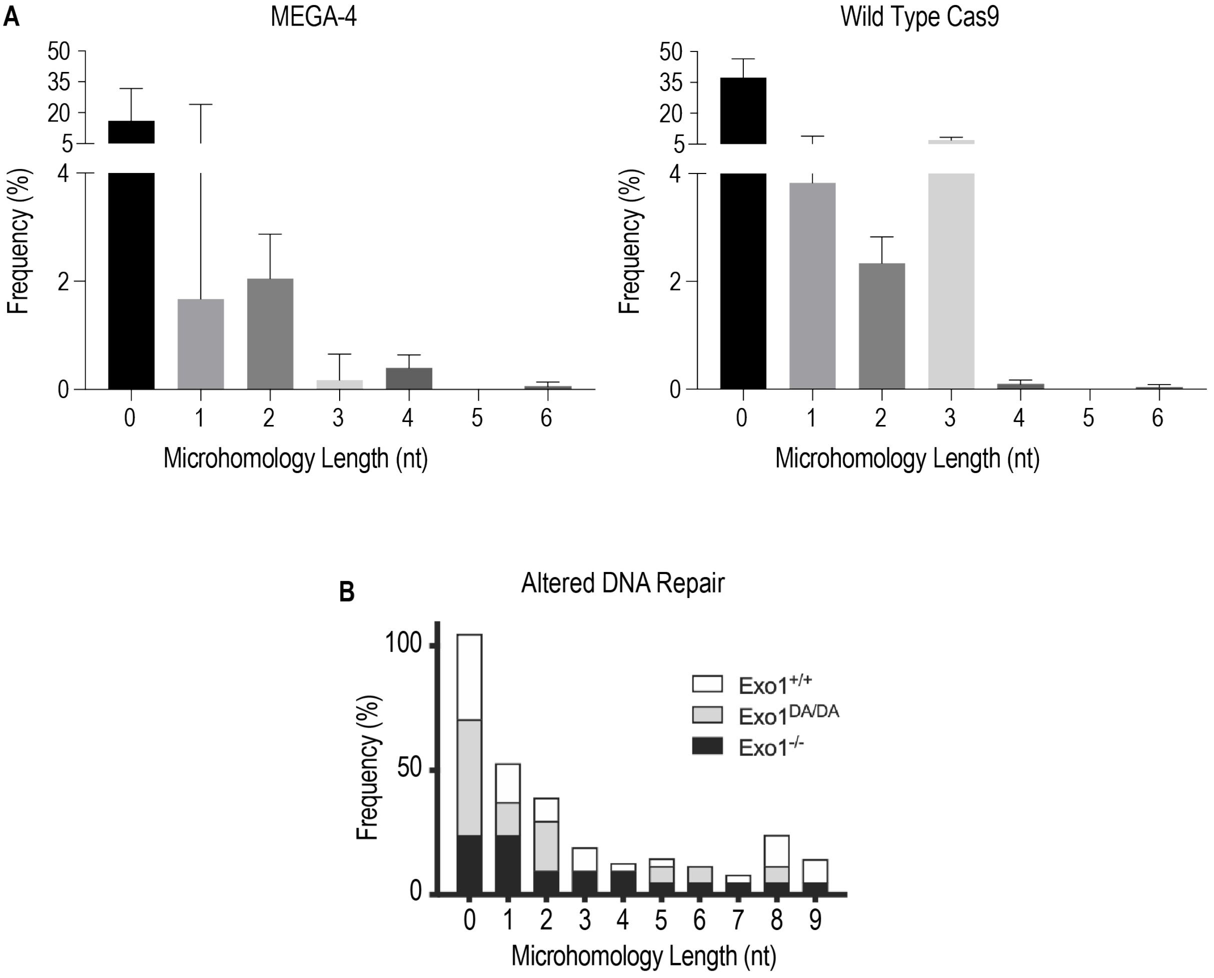

Figure S8: Microhomology length at deletion sites. A) MEGA-4 and wild type Cas9 lead to occurrence of different length microhomologies at DNA break sites. B) Microhomology length depending on exonuclease 1 (Exo1) mouse model (37). $Exo1^{+/+}$ mice (wild type exonuclease 1), $EXO1^{DA/DA}$ mice (nuclease-deficient exonuclease 1), $EXO^{-/-}$ mice (exonuclease 1 null)

A

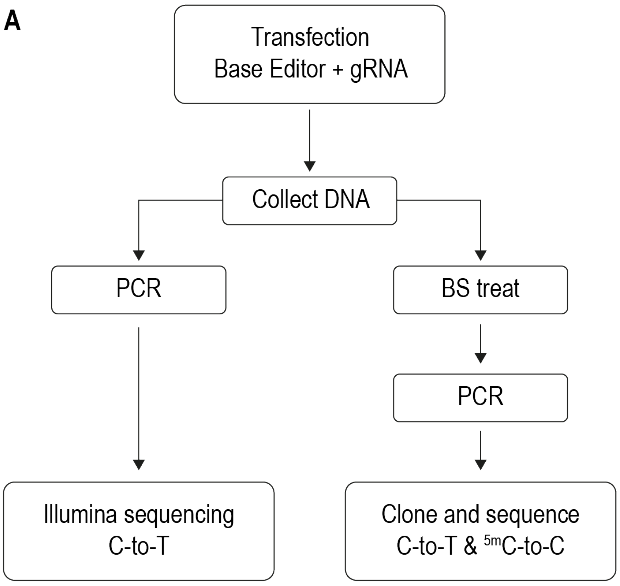

B

Untreated		
5mC Position	N° of Clones w/o Methylation	Total N° of Clones
Position 145	3	6
Position 75	2	7
Position 36	3	7
Position 26	3	7
Position 21	2	7
Position -8	2	7

MEGA-1		
5mC Position	N° of Clones w/o Methylation	Total N° of Clones
Position 145	1	10
Position 75	2	10
Position 36	5	10
Position 26	8	10
Position 21	8	10
Position -8	2	10

MEGA-4		
5mC Position	N° of Clones w/o Methylation	Total N° of Clones
Position 145	2	10
Position 75	4	10
Position 36	3	10
Position 26	6	10
Position 21	5	10
Position -8	5	10

Figure S9: Experimental outline of 5mC demethylation analysis. A) Mouse 3T3 cells were transfected with MEGA-1 or MEGA-4 and MyoD-targeting gRNAs. Genomic DNA was split for two separate analyses. Native genomic DNA was bulk deep sequenced to analyse genomic C-to-T mutations. To evaluate demethylation DNA was bisulphite treated and amplified. Then the amplicons were subcloned and between 6 and 10 random clones were analysed by Sanger sequencing. B) Tables show total number of analysed clones per 5mC position and how many were demethylated. For each condition a separate table is shown.

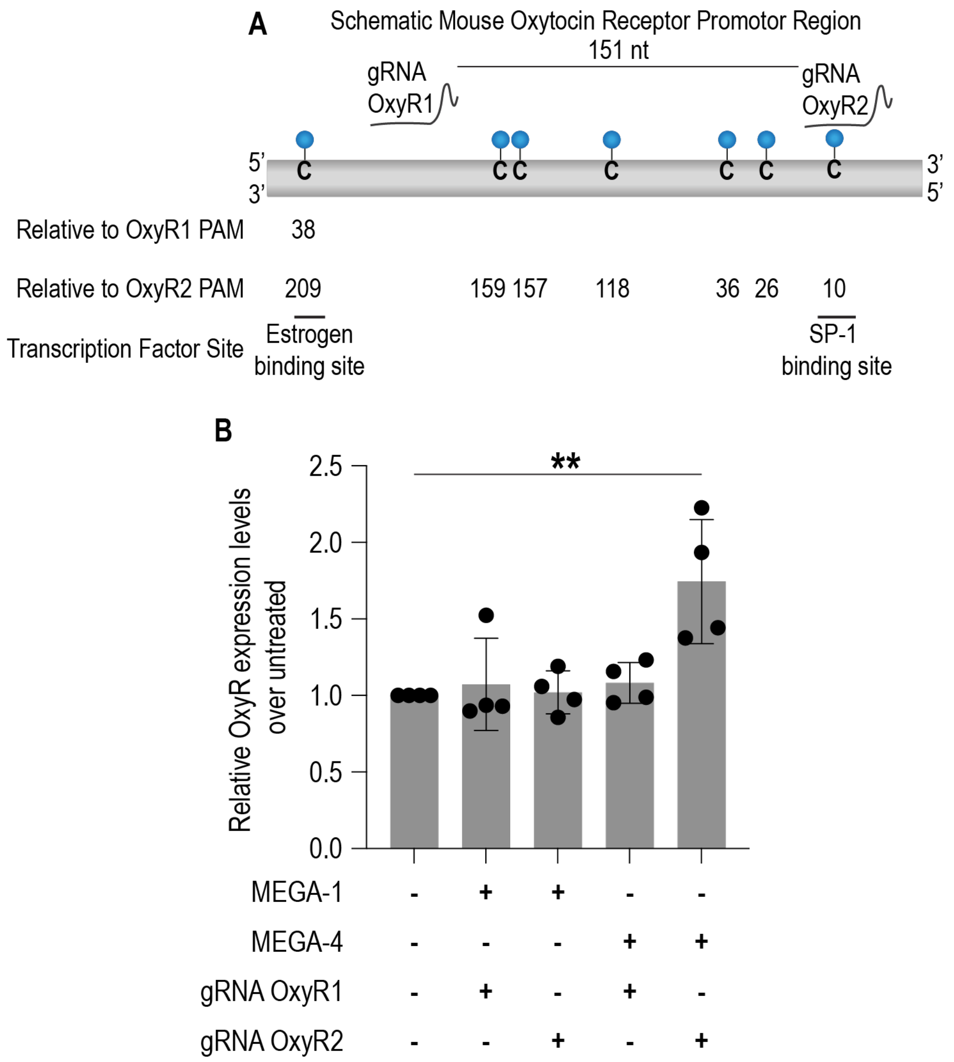

Figure S10: MEGA-4 induces OxyR expression in mouse 3T3 cells. A) Schematic representation of the mouse oxytocin receptor region with the estrogen and SP-1 binding sites. The position of gRNA OxyR1 and OxyR2 are shown. B) Relative OxyR gene expression normalized to housekeeping gene *Actin B* and then normalized to untreated cells. MyoD gene expression was analysed 72h post-transfection by RT PCR. Each dot represents an independent experiment. Standard deviation is shown. Statistical significance was calculated by a one-way ANOVA and multiple comparison (** = $p \leq 0.001$).

Target Gene	gRNA Name	Protospacer (5' – 3')	PAM (5' – 3')
GFP	G1	CAAGCAGAAGAACGGCATCA	AGG
GFP	G2	TACCAGCAGAACACCCCCAT	CGG
GFP	G3	ACGAGGGTGGGCCAGGGCAC	GGG
GFP	G4	GTAGGTCAGGGTGGTCACGA	GGG
GFP	G1'	GATGCCGTTCTTCTGCTTGT	CGG
ATP1A1	A	CATCCAAGCTGCTACAGAAG	AGG
TP53BP1	B	GAACGAGGAGACGGTAATAG	TGG
VH Domain	V1	TGAGTGGATTGGAAATATTA	CCT
VH Domain	V2	CAATGAGAAGTTCAAGAGCA	AGG
VH Domain	V3	TATTGTGCAAGAGATTACTA	CGG
VH Domain	V4	ACTTTGACTACTGGGGCCA	AGG
MyoD Enhancer	MyoD	GCCCAGAGTCAGCTGTTCCT	GGG
MyoD Enhancer Control	Ctrl (MyoD)	CAGCATTTGGGGGCATTTAT	GGG
OxyR2	OxyR	GGGGGTGGGGCGGGGATACA	GGG
OxyR1	OxyR	TCAAAAAGTGATTTCAAAAT	AGG

Table S1: gRNA list

Primer ID	5’-PAD_Sequence-Barcode-Primer_Forward or Reverse-3’
GFP_0001_Forward	GCATC CACTCGACTCTCGCGT CTGAATTCTGACACACCATGGT
GFP_0002_Forward	GCATC TCTGTATCTCTATGTG CTGAATTCTGACACACCATGGT
GFP_0003_Forward	GCATC ACAGTCGAGCGCTGCG CTGAATTCTGACACACCATGGT
GFP_0004_Forward	GCATC ACACTAGATCGCGTGT CTGAATTCTGACACACCATGGT
GFP_0005_Forward	GCATC CGCATGACACGTGTGT CTGAATTCTGACACACCATGGT
GFP_0006_Forward	GCATC CACGACACGACGATGT CTGAATTCTGACACACCATGGT
GFP_0007_Forward	GCATC CACTCACGTGTGATAT CTGAATTCTGACACACCATGGT
GFP_0008_Forward	GCATC CATGTAGAGCAGAGAG CTGAATTCTGACACACCATGGT
GFP_0001_Reverse	GCATC AGAGACTGCGACGAGA CTTGTACAGCTCGTCCATGC
GFP_0002_Reverse	GCATC CAGAGAGTGCGCGCGC CTTGTACAGCTCGTCCATGC
GFP_0003_Reverse	GCATC CGCGCGTCGTCTCAGC CTTGTACAGCTCGTCCATGC
GFP_0004_Reverse	GCATC AGAGAGTACGATATGT CTTGTACAGCTCGTCCATGC
GFP_0005_Reverse	GCATC TCTGTAGTGCGTGCGC CTTGTACAGCTCGTCCATGC
GFP_0006_Reverse	GCATC ATGTGCGTGTGTGTCT CTTGTACAGCTCGTCCATGC
GFP_0007_Reverse	GCATC CTCTCAGACGCTCGTC CTTGTACAGCTCGTCCATGC
GFP_0008_Reverse	GCATC TATCTCAGTGCGTGTG CTTGTACAGCTCGTCCATGC
GFP_0009_Reverse	GCATC TGTGTCTATACTCATC CTTGTACAGCTCGTCCATGC
GFP_0010_Reverse	GCATC TATAGACTATCTGAGA CTTGTACAGCTCGTCCATGC
GFP_0011_Reverse	GCATC GTATGTGAGAGAGCGC CTTGTACAGCTCGTCCATGC
GFP_0012_Reverse	GCATC CACGCGACGCTCTCTA CTTGTACAGCTCGTCCATGC

Table S2 PacBio Sequencing Primers

Primer ID	5'-PAD_Sequence-Primer
GFP_Forward	TCGTCGGCAGCGTCAGATGTGTATAAGAGACAG CTGAATTCTGACACACCATGGT
GFP_Reverse	GTCTCGTGGGCTCGGAGATGTGTATAAGAGACAG GTCTTGTAGTTGCCGTCGTC
TP53BP1_Forward	TCGTCGGCAGCGTCAGATGTGTATAAGAGACAG AAACCGAAAACCAGCAAACAT
TP53BP1_Reverse	GTCTCGTGGGCTCGGAGATGTGTATAAGAGACAG TGACACTAGCACATCAACTTTTG
ATP1a1_Forward	TCGTCGGCAGCGTCAGATGTGTATAAGAGACAG CCCCTCCCACTACTCCTGAA
ATP1a1_Reverse	GTCTCGTGGGCTCGGAGATGTGTATAAGAGACAG GGCATCCCACTTGTAAGAGC
VH_Forward	TCGTCGGCAGCGTCAGATGTGTATAAGAGACAG GCCTGGGACTGAACTGGT
VH_Reverse	GTCTCGTGGGCTCGGAGATGTGTATAAGAGACAG TGTGAGAGTGGTGCCTTGG
Mo_MyoD_Forward	TCGTCGGCAGCGTCAGATGTGTATAAGAGACAG CCACAGCATTTGGGGGCATT
Mo_MyoD_Reverse	GTCTCGTGGGCTCGGAGATGTGTATAAGAGACAG CAGTGCCTTATGGCCATTCC

Table S3 Illumina Sequencing Primers

Primer ID	5’ – 3’ Sequence
Myod1_Mouse_F (Annealing Temp 63 °C)	GGCATGATGGATTACAGCGG
Myod1_Mouse_R (Annealing Temp 63 °C)	GTGGAGATGCGCTCCAC
OxyR_Mouse_F (Annealing Temp 60 °C)	GTGCAGATGTGGAGCGTCT
OxyR_Mouse_R (Annealing Temp 60°C)	GTTGAGGCTGGCCAAGAG
Actb_Mouse_F (Annealing Temp 60/63 °C)	CTAAGGCCAACCGTGAAAAG
Actb_Mouse_R (Annealing Temp 60/63 °C)	ACCAGAGGCATACAGGGACA

Table S4 qPCR Primers
